# Use of medicines for covid-19 treatment in patients with loss of kidney function: a narrative review

**DOI:** 10.1590/2175-8239-JBN-2020-0105

**Published:** 2020-12-11

**Authors:** Lucas Lobato Acatauassu Nunes, Tácio de Mendonça Lima

**Affiliations:** 1 Universidade Federal do Pará, Faculdade de Medicina, Belém, PA, Brasil.; 2 Hospital Universitário João de Barros Barreto, Unidade do Sistema Urinário, Belém, PA, Brasil.; 3 Universidade Federal Rural do Rio de Janeiro, Departamento de Ciências Farmacêuticas, Seropédica, RJ, Brasil.

**Keywords:** Covid-19, Kidney injury, Drug therapy, Dose Adjustment, Covid-19, Insuficiência Renal, Tratamento Farmacológico, Ajuste de Dose

## Abstract

Covid-19 has been identified as the cause of acute respiratory disease with interstitial and alveolar pneumonia, but it can affect several organs, such as kidneys, heart, blood, nervous system and digestive tract. The disease-causing agent (Sars-CoV-2) has a binding structure to the angiotensin-converting enzyme 2 (ACE2) receptor, enabling entry into cells that express ACE2, such as the pulmonary alveolar epithelial cells. However, studies also indicate the possibility of damage to renal cells, since these cells express high levels of ACE2. Currently, there is no evidence to indicate a specific treatment for covid-19. Several drugs have been used, and some of them may have their excretion process altered in patients with abnormal kidney function. To date, there are no studies that assist health professionals in adjusting the dose of these drugs. Thus, this study aims to review and discuss the topic, taking into account factors associated with kidney injury in covid-19, as well as pharmacokinetic aspects and dose recommendations of the main drugs used for covid-19.

## RESUMO

A covid-19 foi identificada como a causa de doença respiratória aguda com pneumonia intersticial e alveolar, mas que pode afetar vários órgãos, como rim, coração, sangue, sistema nervoso e trato digestivo. O agente causador da doença (Sars-CoV-2) tem uma estrutura de ligação ao receptor da enzima de conversão da angiotensina 2 (ACE2), permitindo a entrada em células que expressam ACE2, como as células epiteliais alveolares pulmonares. Porém, estudos também indicam a possibilidade de lesão das células renais, uma vez que essas células expressam altos níveis de ACE2. Atualmente, não existem evidências para a indicação de um tratamento específico para a covid-19. Vários medicamentos vêm sendo utilizados, e alguns podem ter o processo de eliminação alterados em pacientes com comprometimento renal. Até o momento, não há estudos que auxiliem os profissionais de saúde no ajuste de dose desses medicamentos. Assim, este estudo tem como objetivo revisar e discutir o tema, levando em consideração os fatores relacionados à lesão renal na covid-19, bem como aspectos farmacocinéticos e recomendações de doses dos principais medicamentos utilizados para covid-19.

## INTRODUCTION

Coronaviruses are important human and animal pathogens. In late 2019, a new coronavirus called covid-19 was identified as the cause of a set of pneumonia cases in Wuhan, a city in China's Hubei province. It spread rapidly, resulting in an epidemic across China, followed by an increasing number of cases in other countries in the world, leading to a pandemic.^1^

Transmission occurs from person to person, mainly through respiratory droplets. The virus is released through respiratory secretions when an infected person coughs, sneezes or speaks, and can infect another person if it comes in direct contact with mucous membranes; infection can also occur if a person comes into contact with an infected surface and then touches the eyes, nose or mouth.^2^

The causative agent of covid-19 (Sars-CoV-2) is of the same subgenus as the Sars virus, with a binding structure to the angiotensin-converting enzyme 2 (ACE2) receptor that allows entry into cells.^3^ The pulmonary parenchyma has abundant ACE2 receptors in its alveolar epithelial cells, manifesting mainly as an acute respiratory disease with interstitial and alveolar pneumonia, but it can affect several organs, such as kidneys, heart, blood, nervous system and digestive tract.^4^^,^ 5

An *in vitro* study with renal proximal tubular epithelial cells established that Sars-CoV manifested persistent and productive infection, which was partially correlated with ACE2.^6^ expression using state-of-the-art single-cell techniques, Zou and colleagues showed high stratified organs and low risk, according to the level of ACE2 expression, indicating the kidneys as high-risk organs.^7^ These findings indicate the possibility of Sars-CoV-2 infecting renal cells.

On the other hand, drugs with potential efficacy against covid-19 have been increasingly studied and used in diagnosed patients, especially in the hospital environment.^8^ We know that the metabolism and clearance of many drugs depend on normal kidney function. These drugs can be altered by various pharmacokinetic processes in situations of kidney function deficit, subsequently resulting in adverse effects and clinical signs of drug intoxication in these patients.^9^^,^ 10

To date, there are no studies that assist health professionals in adjusting the dose of drugs used to treat covid-19 in patients with loss of kidney function. Thus, the aim of this study is to discuss the complexity of the topic, taking into account factors associated with the pathophysiology of acute kidney injury (AKI) and kidney abnormalities in covid-19, as well as pharmacokinetic aspects and recommendations for doses of the main drugs used for covid-19.

## METHODS

This is a narrative review of the literature. We ran a quick search for papers indexed in the Medline databases (PubMed) and the Virtual Health Library (VHL), using keywords or combinations of the terms "covid-19", "kidney injury" and "drug treatment" . Preprint servers, such as medRxiv and SciELO Preprints, were also used in search of manuscripts of interest on the topic and not yet published in scientific journals. Current and relevant studies with the theme were chosen to make up the body of the review. In addition, the established literature^9^^,^ 11 related to pharmacokinetic aspects in patients with loss of kidney function was also used.

The drugs included in this review were selected based on the guidelines for diagnosis and treatment of covid-19 from the Ministry of Health of Brazil,^12^ on the panel of guidelines for treatment of covid-19 from the National Institutes of Health^13^ and recommendations on treatment evidence for covid-19 from the American Society of Health-System Pharmacists.^14^ Information on the use of these drugs in the treatment of covid-19 was analyzed in information bases on prestigious drugs, such as Uptodate^®^,^15^ Micromedex^®^ 16 and The Sanford Guide,^17^ as well as in relevant literature^10^^,^ 18 in the theme field.

## ACUTE KIDNEY INJURY IN COVID-19

The pathogenesis of acute kidney injury associated with covid-19 is multifactorial, but volume depletion on admission, secondary to fever and insensitive losses, can be a common trigger for AKI, since pre-hospital resuscitation with fluids is rarely performed, leading to a pre-renal AKI due to renal hypoperfusion.^19^ Rhabdomyolysis may also be associated with AKI, since critically ill patients commonly present significant elevations of creatinophosphokinase (CPK). A previous study^20^ carried out with kidney biopsy identified hemosiderin granules with pigmented cylinders in the epithelium of the renal tubules.

Direct lesion of the renal tubular epithelium and podocytes has already been described by electron microscopy, identifying viral particles in the cytoplasm of the proximal tubular epithelium and in the podocytes, through a pathway dependent on the angiotensin-converting enzyme (ACE2) receptor, causing mitochondrial dysfunction, acute tubular necrosis (ATN).^20^^,^ 21 The presence of proteinuria and hematuria in routine urinalysis can be related to glomerular lesions, ranging from secondary erasure of the pedicels to the presence of glomerular collapses (probably the most common glomerular disease) associated with the APOL1 gene.^21^^,^ 22 ^,^ 23

Elevated levels of inflammatory cytokines can participate in AKI, interacting with cells residing in the kidneys and inducing endothelial and tubular dysfunction. For example, TNF-α can bind directly to tubular cell receptors, triggering the apoptosis death receptor pathway.^22^^,^ 24 Other studies have also found an increase in IL-6 in critically ill patients with covid-19 in the group that evolved to death in patients with covid-19 when compared to survivors.^23^^,^ 25

Infection with covid-19 can also target lymphocytes, since they express the angiotensin-converting enzyme 2 (ACE2), leading to lymphocyte activation and, consequently, cell death induced by this activation, which can result in lymphopenia. In addition, procoagulation pathways and complement systems can activate each other, as seen in the deposition of complement C5b-9 (membrane attack complex) in the renal tubules in a study of six patients infected with Sars-CoV- 2, suggesting activation of the complement pathway.^26^ This deregulation of the immune response related to covid-19 may predispose to the state of hypercoagulability, since innate immunity and coagulation pathways are closely linked.^27^ Activation of macrophages associated with covid- 19, hyperferritinemia, cytokine storm and the activation of molecular proteins associated with damage can result in the release of tissue factor and the activation of coagulation factors, predisposing fibrin deposits in the renal glomerular loops,^28^ leading to a predisposition to hypercoagulability, which may contribute to renal microcirculatory dysfunction and AKI.

AKI has been reported as one of the complications that occur during the progression of covid-19, both in patients with previous kidney disease and in those who do not have it.^29^^,^ 30 The glomerular filtration rate evaluation should be in accordance with the guidelines in force for AKI.^28^ Therefore, kidney function should be monitored in a day-by-day basis, as until now, there has been no pattern of kidney function alteration in these patients. Zhou et al^31^ reported that AKI appears to develop on average 15 days after hospital admission, while other studies mention the development of AKI on an average of 5-7 days of admission.^32^^,^ 33

The incidence of AKI in patients with covid-19 is not consistent and has varied, on average, from 0.5% to 23%, occurring mainly in patients who already had changes in kidney function upon hospital admission and in critically ill patients who need intensive care.^5^^,^ 31 ^,^ 33

From the current epidemic, data on AKI and onset of RRT are related to increased mortality, similar to a study on Sars-CoV infection, in which AKI developed in 6.7%, with a well-established mortality rate. higher in these patients (91.7%), versus 8% in those who did not develop AKI.^32^^,^ 33 ^,^ 34 However, it is not yet possible to describe whether only AKI is an independent risk factor for mortality or if the group with AKI has the sickest patients, most likely to die due to severe clinical condition.^31^^,^ 33 ^,^ 34

## PHARMACOKINETIC ASPECTS IN LOSS OF KIDNEY FUNCTION

Pharmacokinetic processes are altered in patients with impaired kidney function, such as bioavailability, volume of distribution, biotransformation and excretion.^10^

### BIOAVAILABILITY

The bioavailability of a drug represents the percentage of a given dose of a drug available in the systemic circulation.^9^^,^ 11 It is basically determined by the speed and route of administration, but it also depends on the intensity of absorption and pre-systemic metabolism, hepatic or pulmonary effects of drugs (first-pass effect). We consider 100% bioavailability when the drug is administered intravenously.^9^^,^ 10

Bioavailability may be altered in patients with abnormal kidney function. Regarding the first-pass effect, bioavailability may increase for certain drugs, because of an intrinsic reduction in hepatic metabolism. For others, it may decrease due to the increased release of uremic factors, such as parathyroid hormone and inflammatory cytokines, resulting in an alkaline gastric environment.^9^^,^ 10

Changes in motility and gastrointestinal absorption are also significant causes of reduced drug bioavailability. Nausea, vomiting, diarrhea and gastroparesis, common in uremic patients, can alter motility and decrease absorption. In addition, these patients commonly present with edema of the intestinal wall and increased gastric pH due to increased production of ammonia by the action of urea, compromising the absorption of medications.^9^^,^ 10

### VOLUME OF DISTRIBUTION

The volume of distribution (Vd) of a drug reflects the extent to which it is present in extravascular tissues, except in the plasma.^11^ It depends on the degree of binding of drugs to tissues and proteins and on their liposolubility; liposoluble drugs or those that bind widely to body tissues generally have higher Vd, unlike drugs that bind more to plasma proteins.^9^

There is an inverse correlation between serum concentration and Vd. Thus, changes in the volume of extracellular fluid can affect Vd.^10^ In patients who lost kidney function, the drug's Vd may increase due to edema and ascites, mainly with water-soluble drugs, resulting in a lower serum concentration of it. On the other hand, uremic patients may develop hypoproteinemia, especially hypoalbuminemia, and circulating organic residues that bind to carrier proteins and displace drugs from their binding to plasma proteins, resulting in high free and active fractions of these drugs at usual doses and, consequently, possible toxicity.^9^^,^ 10

### BIOTRANSFORMATION AND EXCRETION

Biotransformation or metabolism consists of the biochemical conversion of a drug that has a given chemical characteristic into another compound (metabolite) with a distinct chemical characteristic. Most biotransformation occurs in the liver, through hepatic metabolism pathways, including oxidation, reduction, acetylation and hydrolysis. The result is a more polar and hydrophilic metabolite, which is more easily excreted.^10^ In uremic patients, hepatic drug metabolism can reduce, especially reduction, acetylation and hydrolysis.^9^ This is due to the presence of uremic toxins that can inactivate cytochrome P450 enzymes.^10^

Regarding the excretion of drugs unchanged or in the form of metabolite (active or not), patients with abnormal kidney function can lead to the body buildup of these compounds and prolongation of their actions in the body.

Regarding the excretion of the drugs unchanged or in the form of metabolite (active or not), patients with abnormal kidney function can have a body buildup of these compounds and prolongation of their actions in the body, resulting in a possible toxicity.^10^

## MEDICINES USED FOR COVID-19 TREATMENT

To date, there is no high-quality evidence that makes it possible to indicate a specific drug therapy for covid-19.^12^ several therapeutic alternatives have been used, including chloroquine, hydroxychloroquine, azithromycin, antiparasitic, corticosteroids, heparins, tocilizumab and antivirals. It is important to note that these drugs have been used off-label in Brazil, or have not yet been registered with the National Health Surveillance Agency (Anvisa) for commercialization, such as favipiravir and remdesivir. On the other hand, clinical studies are being conducted in Brazil and worldwide in order to evaluate the efficacy and safety of these drugs for the treatment of covid-19.

In this context, we know that some of these drugs are excreted unchanged by the kidneys, or their metabolites, and may increase the risk of adverse reactions and toxicity in patients with abnormal kidney function.^35^ On the other hand, for many drugs, some or even all altered pharmacokinetic parameters are unknown. In such circumstances, professional judgment should be used to predict the distribution of these drugs in the body, based on knowledge of the chemical structure of the drug, its class and pharmacokinetics in patients with normal kidney function.^15^ In addition, kidney function generally decreases with age (population most affected by covid-19), and many elderly people have a Glomerular Filtration Rate (GFR) of less than 50 mL/min which, due to reduced muscle mass, may not be reflected by an elevated creatinine.^18^

The following are the main drugs used to treat covid-19 according to the main guidelines and recommendations from Brazil and the world, their main pharmacokinetic characteristics (Table 1), as well as dose recommendations and possible dose adjustments for these drugs (Table 2).

**Table 1:** Main pharmacokinetic chracteristics^15^^,^ 16 ^,^ 18 of the drugs used to treat covid-19

Drugs	Molecular weight (Da)	Bioavailability (%)	PP binding (%)	Unaltered renal excretion (%)	Volume of distribution (L/kg)	Half-life
Chloroquine	319,9	67 - 114	50 - 70	42 - 47	132	10-60 days
Hydroxychloroquine	434	67 - 74	30 - 40	3	ample	172,3 hours -50 days
Azithromycin	785	37	15 - 52	6 - 12	31,1	48-96 hours
Nitazoxanide	307,3	70	> 99 (tizoxanide)	33	NR	6 minutes; 1,0-1,6 hours (tizoxanide)
Ivermectin	875,1	60*	93 (albumin)	< 1	46,8	18 hours
Dexamethasone	392,5	100	77	65	0,8 - 1,0	3,5-4,5 hours
Methylprednisolone	375	100	77	< 10	1,4	1,8-5,2 hours
Tocilizumab	148.000	80 - 96	NR	NR	6,4	11-13 days
Unfractionated heparin	3.000 - 30.000	NR	> 90 (LDL)	0	0,06 - 1	1,5 hours
Enoxaparin	4.500	~ 100	80	10	4,3	4-5 hours
Lopinavir/Ritonavir	628,8	**	98 - 99	2,2	0,5	5-6 hours
Oseltamivir	410,4	75	42	99 (carboxylated metabolite)	0,3 - 0,4	1-3 hours
Favipiravir	157	97,6	54 (albumin)	0,8	15 - 20	2-5,5 hours
Remdesivir	602,5	NR	NR	NR	NR	20 hours (metabolite)

Legend: LDL (low-density lipoprotein); NR (not reported); PP (plasma proteins)

* Use with food increases availability 2.5 fold.

** Lopinavir has low bioavailability (~25%), significantly increasing when coadministered with ritonavir. Use with food increases availability in ~19%.

**Table 2:** Dose recommendations and possible adjust of the drugs used to treat covid-19

Drugs	Recommended doses^12^^,^ 13 ^,^ 14 ^,^ 15 ^,^ 16 ^,^ 17 ^,^ 40	Dose adjustment (GFR mL/min)^15^^,^ 16 ^,^ 18			Dialysis^15^^,^ 16 ^,^ 18			
		50-31	30-10	< 10	APD/CAPD	HD	HDF/AF	CAV/VVHD
Chloroquine	Day 1: 450 mg bid PO Days 2 to 5: 450 mg once per day PO	Usual dose	Usual dose	50% of the dose	ND	ND	NR	ND
Hydroxychloroquine	Day 1: 400 mg bid PO Days 2 to 5: 400 mg once per day PO	Usual dose	50% of the dose	50% of the dose	ND	ND	NR	NR
Azithromycin	500 mg once per day PO for 5 days	No adjustment			ND	NR	NR	NR
Nitazoxanide	600 mg bid PO for 5 days	No adjustment			NR	NR	NR	NR
Ivermectin	200 mcg/kg single dose, can be repeated on day 7	No adjustment			NR	NR	NR	NR
Dexamethasone	6 mg once per day PO or IV for 10 days	No adjustment			ND	ND	NR	I
Methylprednisolone	1-2 mg/kg once per day IV for 5-7 days	No adjustment			D (dose usual)	D (dose usual)	D (dose usual)	D (dose usual)
Tocilizumab	8 mg/kg (maximum of 800 mg) IV single dose, can be repeated 1-2 times if there is no clinical improvement within 24 hours	No adjustment			NR	NR	NR	NR
Unfractionated heparin	10.000-15.000 U once per day	No adjustment			ND	ND	ND	ND
Enoxaparin	P: 40 mg once per day* SQ T: 0,5-1 mg/kg bid SQ	Usual dose	P: 20-30 mg/day T: 0,5-1mg/kg/day	P: 20-30 mg/day T: 0,5-1mg/kg/day	ND	ND	D (dose GFR < 30)	ND
Lopinavir/ Ritonavir	400 mg/100 mg once per day PO for 10-14 days	No adjustment			L: I R: ND	L: I R: ND	L: I R: ND	L: NR R: I
Oseltamivir	75 mg bid PO for 5 days	30 mg bid	30 mg/day	30 mg single dose	D (30 mg)	D (30 mg)	D**	D (30 mg)
Favipiravir	Day 1: 1600 mg bid PO Days 2 to 7 to 14: 600 mg bid PO	No adjustment			NR	NR	NR	NR
Remdesivir	Day 1: 200 mg once per day IV Days 2 to 5 or 10: 100 mg once per day IV	Usual dose	Not recommended	Not recommended	NR	NR	NR	NR

Legends: APD/CAPD (automated peritoneal dialysis/continuous ambulatory peritoneal dialysis); bid (twice a day); CAV/VVHD (continuous arteriovenous hemofiltration/veno-venous hemofiltration); D (dialyzed); GFR (glomerular filtration rate); HD (intermittent hemodialysis); HDF/AF (intermittent hemodialysis/high flow); PO (oral administration); I (improbable); IV (intravenous administration); L (lopinavir); ND (not dialyzed); NR (not reported); P (prophylaxis); R (ritonavir); SQ (subcutaneous administration); T (treatment).

* Body Mass Index (BMI) ≥ 40 kg/m2: increase the dose in about 30%; or 0.5 mg/kg/day using current weight for the calculation.

** Flow Rate 1-1.8 L/hr: 30 mg/day, flow rate 1.9-3.6 L/hr: 30 mg bid, flow rate > 3.6 L/hr: 75 mg bid.

### CHLOROQUINE AND HYDROXYCHLOROQUINE

About 50% of chloroquine is excreted unchanged, and 10% as a metabolite by the kidneys, which can lead to its buildup in the body, in addition to further prolonging the drug's half-life, which is already high (10-60 days).^18^ Therefore, a 50% dose reduction is recommended in patients with GFR < 10 mL/min. Regarding patients receiving dialysis, there is no recommendation to supplement the dose for most existing dialyses.^8^^,^ 18 Hydroxychloroquine is only 3% excreted unchanged, but it is metabolized to chloroquine and active metabolites that can also buildup in patients with kidney injury. Thus, a 50% reduction is recommended in patients with GFR < 30 mL/min. There is no need to supplement the dose of these drugs for any of the existing dialysis treatments.^18^

### AZITHROMYCIN

There is no recommendation to adjust the dose of azithromycin in patients with abnormal kidney function. However, it is important to use it with caution in patients with GFR < 10 mL/min, as it can increase adverse gastrointestinal effects, such as diarrhea, nausea and vomiting due to the increase (around 33%) of the systemic exposure of azithromycin.^15^^,^ 18 Furthermore, as azithromycin is associated with hydroxychloroquine in the treatment of covid-19, and both may be accumulating in the body of these patients, effective electrocardiogram monitoring must be performed due to the greater risk of QT interval prolongation.^15^^,^ 16 ^,^ 17 It is recommended that azithromycin be administered within 4 hours of hydroxychloroquine. There is no need to supplement the dose for any of the existing dialysis treatments.^18^

### ANTIPARASITIC

There are no dose adjustments provided by the manufacturer for nitazoxanide. However, this drug is excreted unchanged by the kidneys by up to 33%, and can be accumulated in the body of patients with abnormal kidney function, as well as its active metabolite (tizoxanide), which is highly linked to plasma proteins (> 99%), being able to be displaced, raising the free fractions in these patients.^15^ In addition, a study carried out in rats showed a significant increase in the serum level of creatinine and urea one day after treatment with nitazoxanide, when compared with the control group.^36^ it results in the use of this medication in patients with abnormal kidney function.^17^ There are no studies that support the need for dose supplementation in any of the dialysis treatments used.^17^^,^ 18

There are also no dose adjustments provided by the manufacturer for Ivermectin. However, this drug is highly linked to plasma proteins (93%), mainly albumin, and can be displaced, increasing free fractions in patients with loss of capacity.^15^^,^ 16 In addition, a study carried out in rats showed that the use of Ivermectin can compromise kidney and liver integrity.^37^ There are no studies that support the need for dose supplementation for any of the existing dialysis treatments.^17^^,^ 18

### CORTICOSTEROIDS

There is no recommendation to adjust the dose in patients with abnormal kidney function, although 65% of dexamethasone is excreted unchanged within 24 hours. However, the use of corticosteroids should be done with caution in patients with loss of kidney function, as greater fluid retention may occur. Dexamethasone should also be used with caution in the elderly, always in the lowest possible dose.^15^^,^ 16 There is no need to supplement the dexamethasone dose for any of the existing dialysis treatments. On the other hand, patients using methylprednisolone should receive the usual doses after dialysis treatment.^18^

### TOCILIZUMAB

There is no recommendation for dose adjustment in patients with abnormal kidney function. However, this drug has a high molecular weight (148 kDa), and it is unlikely to be significantly eliminated by the renal route in patients with GFR < 30 mL/min. Thus, we recommend caution regarding its use and monitoring of kidney function in these patients.^15^^,^ 16 There are no studies that support the need for dose supplementation for any of the existing dialysis treatments.^18^

### ANTICOAGULANTS

There is no recommendation to adjust the dose of unfractionated heparin in patients with abnormal kidney function. However, there may be greater renal elimination (around 50%) at high doses, in addition to an increase in the drug's half-life.^15^^,^ 16 ^,^ 18 Therefore, caution should be exercised when using it in high doses. There is no need to supplement the dose for any of the existing dialysis treatments.^18^

In contrast, enoxaparin needs dose adjustment in these patients, since there is a risk of bleeding due to decreased renal clearance (about 30%) and increased bioavailability. In addition, although 10% of the drug is excreted unchanged by the kidney, a large part of the active and inactive metabolites (around 40%) is excreted by this route and can accumulate in these patients. Finally, 80% of the drug is bound to plasma proteins, being able to be displaced and increasing free fractions in patients with loss of kidney function.^15^^,^ 16 Therefore, a dose reduction to 20 to 30 mg/day is recommended (prophylactic ) or 0.5-1 mg/kg/day (treatment) in patients with GFR < 30 mL/min.^15^^,^ 38 Regarding the existing dialysis treatments, there is no need for dose supplementation, except for intermittent hemodiafiltration, in which it is recommended to administer an additional dose similar to the dose used in patients with GFR < 30 mL/min.^18^

### ANTIVIRALS

There are no dose adjustments for lopinavir/ritonavir provided by the manufacturer, and a reduction in the clearance of these drugs is unlikely in patients with kidney injury. However, these drugs are highly bound to plasma proteins (> 98%) and can be displaced, increasing free fractions in these patients.^15^^,^ 16 It should also be used with caution in elderly patients, since the data on these patients are insufficient to determine whether they respond differently from adults.^39^^,^ 40 ^,^ 41 There is no need for dose supplementation for any of the existing dialysis treatments.^18^

Oseltamivir is a prodrug, extensively metabolized by esterases in the liver to the active carboxylate metabolite. This metabolite is eliminated by renal excretion (99%). Furthermore, renal clearance exceeds the glomerular filtration rate, indicating that tubular secretion occurs in addition to glomerular filtration.^15^^,^ 18 This metabolite can accumulate in the body of patients with abnormal kidney function. Thus, a dose of 30 mg twice daily is recommended for patients with GFR between 31 and 60 mL/min; 30 mg daily to patients with GFR between 10 and 30 mL/min; and 30 mg single dose to patients with GFR < 10 mL/min.^17^^,^ 18 In patients undergoing hemodialysis and peritoneal dialysis, an initial dose of 30 mg can be administered before starting dialysis and supplemented with 30 mg each session or 5 days, respectively.^18^ Continuous or venous arteriovenous hemofiltration is similar to hemodialysis. For intermittent hemodiafiltration, the doses indicated will depend on the flow rate, which is detailed at the bottom of Table 2.^18^

For favipiravir, there are no dose adjustments provided by the manufacturer, because available data is limited.^17^ However, this drug undergoes hepatic metabolism, generating metabolites that are excreted in renal hydroxylated forms. The fraction of metabolites excreted in the urine increases over time, reaching 80-100% after 7 days.^42^ Thus, it is important to monitor patients with loss of kidney function, as well as the elderly. There are no studies that support the need for dose supplementation for any of the existing dialysis treatments.^17^

Finally, there are no studies recommending dose adjustment of remdesivir in patients with loss of kidney function, as there are no safety or pharmacokinetic data available for this population. However, animal studies have shown an increase in urea and mean creatinine, as well as renal tubular atrophy in histopathological findings, indicating altered kidney function;^43^ therefore, use in patients with GFR < 30 mL/min is not recommended.^8^ Patients receiving Kidney Replacement Therapy (KRT) were excluded from current clinical trials.^15^

## CONCLUSION

The topics presented in this study show that patients who have abnormal kidney function and were affected by covid-19 undergo several physiological changes that can cause changes in the pharmacokinetics and pharmacodynamics of drugs, which can cause variations in their serum concentrations and, consequently, risk of overdosing and toxicity.

In addition, many drugs used to treat covid-19 require dose adjustments or continuous monitoring in this population.

Thus, the indication and use of these drugs must be well evaluated, checking the risk-benefit of the therapy, taking into account the particularities of each patient.
